# SAAM-VetNet: an attention-based multi-task framework for animal disease detection and severity grading

**DOI:** 10.1097/MS9.0000000000003728

**Published:** 2025-08-19

**Authors:** Ishana Attri, Brij Vanita, Rajesh Rajput, Lalit Kumar Awasthi, Ankaj Thakur, Deepraj Tripathi, Virender Pathak, Parul Shukla, Divya Gupta

**Affiliations:** a School of Computer Science and Engineering, Galgotias University, Uttar Pradesh, India; b DGCN College of Veterinary & Animal Sciences, Chaudhary Sarwan Kumar Himachal Pradesh Krishi Vishvavidyalaya, Palampur, Himachal Pradesh, India; c Department of Computer Science and Engineering, NIT Hamirpur, Himachal Pradesh, India; d Department of Applied Sciences, IIIT Allahabad, Uttar Pradesh, India

**Keywords:** Animal disease detection, Attention mechanism, Deep learning, Preclinical model, Severity grading, Veterinary diagnostics

## Abstract

Early and accurate detection of animal diseases is critical in veterinary medicine and preclinical research, where timely intervention can influence both animal welfare and experimental outcomes. In this study, we introduce SAAM-VetNet, a novel Severity-Aware Attention-Based Multi-Task deep learning framework designed to simultaneously detect animal diseases and grade their severity from medical images. The proposed architecture integrates a convolutional block attention module to enhance feature localization and contextual representation, coupled with a multi-branch learning strategy for disease classification and severity assessment. We evaluate SAAM-VetNet using two publicly available datasets: the Animal Disease Classification dataset and the Mastitis Disease Detection dataset. Our model achieves superior performance with an accuracy of 91.2% and an *F*1 score of 89.8%, outperforming established baselines including ResNet18, MobileNetV2, EfficientNet-B0, DenseNet121, and vision transformer (ViT). The results demonstrate that incorporating attention mechanisms and severity-aware multi-task learning significantly enhances model interpretability and diagnostic accuracy, offering a robust tool for automated preclinical model selection and veterinary diagnostics.

## Introduction and background

Preclinical surgical research forms a vital component of translational medicine, enabling researchers to test surgical techniques, devices, and treatment protocols in controlled environments before human application. The reliability of such studies depends heavily on the health and consistency of the animal models used. The early and accurate detection of animal diseases is a cornerstone of effective veterinary care and an essential component of preclinical biomedical research. In laboratory and agricultural settings, delayed or inaccurate diagnosis can lead to unnecessary suffering, economic loss, and compromised research validity. Despite the critical nature of this task, conventional diagnostic approaches are often manual, labor-intensive, and subject to observer bias. This has spurred interest in developing automated systems that can support or replace human judgment through the use of computer vision and deep learning techniques.

**HIGHLIGHTS**

- SAAM-VetNet integrates convolutional block attention module attention and multitask learning for automated veterinary diagnostics.
- Achieves 91.2% classification accuracy and 89.8% *F*1 score on animal disease datasets.
- Simultaneously predicts disease class and severity, enhancing clinical decision-making.
- Outperforms ResNet18, MobileNetV2, EfficientNet-B0, DenseNet121, and vision transformer.
- Offers robust, interpretable insights for preclinical model selection and animal health monitoring.

Recent advances in convolutional neural networks (CNNs) and vision transformers (ViTs) have demonstrated considerable promise in medical and veterinary imaging tasks. However, two major challenges remain under-addressed: (1) the accurate localization of disease-relevant features in complex biological imagery and (2) the assessment of disease severity, which is crucial for clinical decision-making and the selection of appropriate preclinical models. Most existing systems are limited to binary or multiclass classification without considering the gradation of disease expression, which is essential in real-world diagnostic contexts.

To address these limitations, we propose SAAM-VetNet, a Severity-Aware Attention-Based Multi-Task deep learning framework for the automated detection and grading of animal diseases from image data. Our model incorporates a convolutional block attention module (CBAM) to refine spatial and channel-wise attention, thereby enhancing the network’s ability to focus on diagnostically relevant regions. Furthermore, we adopt a multi-task learning strategy that enables simultaneous disease classification and severity scoring, improving both performance and interpretability.

We evaluate SAAM-VetNet on two real-world datasets: the Animal Disease Classification dataset, which includes a variety of infectious conditions, and the Mastitis Disease Detection dataset, which targets a common inflammatory disease in dairy animals. Compared to several baseline architectures – including ResNet18, MobileNetV2, EfficientNet-B0, DenseNet121, and the ViT – our proposed model demonstrates superior performance in both accuracy and *F*1 score.

In the domain of related work, deep learning continues to revolutionize veterinary diagnostics across species and disease types. Girmaw demonstrated 99.01% accuracy in skin disease classification in livestock using EfficientNetB7^[1]^. Chu *et al* employed a multi-scale attention-augmented DenseNet architecture to assess mastitis severity from thermal images, achieving 90.18% accuracy^[2]^. Ismail *et al* addressed lameness detection using raw sensor data in a behaviorally grounded architecture, LLP-Cow, achieving 0.94 precision and 0.98 specificity^[3]^. Cihan *et al* advanced cardiovascular disease diagnosis in cattle through retinal imaging, with ResNet101 attaining 96.1% accuracy^[4]^. Ruchay *et al* explored cattle facial recognition via transfer learning using VGGFACE2, achieving 97.1% recognition accuracy^[5]^. Takahashi *et al* applied transfer learning to assess teat-end hyperkeratosis, an early indicator of mastitis, achieving 80–86% classification accuracy^[6]^. Freitas *et al* developed a novel method for diagnosing anemia in sheep using ocular conjunctiva images processed via U-Net segmentation and classified using VGG19^[7]^.

Recent advances in deep learning have also significantly expanded its role in veterinary diagnostic imaging, health monitoring, and food safety. In veterinary diagnostic imaging, attention mechanisms and self-supervised learning frameworks such as VET-DINO have demonstrated enhanced anatomical understanding using multi-view radiographs for improved feature extraction^[8]^. Transformer-based architectures have shown promise in veterinary diagnostics, providing nuanced data interpretation across diverse modalities^[9]^. In severity grading, advanced methods such as ConPro leverage contrastive learning with preference optimization for severity estimation^[10]^, while SEMISE integrates self-supervised and supervised learning for more robust severity representation^[11]^.

Artificial intelligence (AI)-powered tools have also shown broad applicability across veterinary disease detection domains. Ott *et al* developed CNN-based models to detect pulmonary Coccidioidomycosis (Valley Fever) in canines with an AUC of 0.99^[12]^, while Buric *et al* applied U-Net-based segmentation for canine ophthalmologic disease detection^[13]^. In musculoskeletal and organ imaging, Agrawal and Kumar introduced LiDSCUNet++, a lightweight UNet++ architecture combined with YOLOv8 for vertebral segmentation and spondylosis detection in dogs^[14]^. Recently, Choi *et al* proposed PFSH-Net, a parallel frequency-spatial hybrid network for automatic segmentation of kidney stones in canine CT scans, addressing multi-scale lesion size challenges in veterinary urological imaging^[15]^.

Vickram *et al* reviewed AI applications in veterinary imaging and surgery^[16]^, while Burti *et al* emphasized ethical considerations and human–AI collaboration in clinical decision support^[17]^. Additionally, Appleby and Basran provided a comprehensive review outlining the current applications, opportunities, and challenges of AI integration in veterinary diagnostic imaging, emphasizing areas such as image quality improvement, workflow optimization, radiomics, and computer-aided diagnosis^[18]^.

Beyond imaging, explainable AI is being applied in veterinary food safety and public health. Dong *et al* combined hyperspectral imaging with CNN-SSAE and SHAP values for detecting veterinary drug residues in mutton^[19]^. Vázquez *et al* proposed an energy-efficient hybrid spiking neural network for detecting *Eimeria* parasites in poultry and rabbits^[20]^, while Sulthana *et al* developed a lightweight deep CNN (S-MobileNet) for highly accurate multi-class skin lesion classification^[21]^. In livestock management, Rodríguez Alvarez *et al* applied CNNs to estimate body condition scores in cows using depth images^[22]^.

Deep learning has also contributed to behavioral and ecological research. Fazzari *et al* provided a detailed survey of AI-based animal behavior analysis approaches^[23]^, while Saoud *et al* reviewed deep learning techniques for animal movement tracking and multi-animal behavior segmentation^[24]^. In aquaculture, Zakaria *et al* introduced a deep learning-based framework for shrimp disease detection through gut microbiome modulation^[25]^.

Recent literature underscores the growing convergence of AI with veterinary anatomy, diagnostics, and surgical modeling, reinforcing its transformative potential in preclinical research. Choudhary *et al*^[26]^ emphasized that the selection and maintenance of animal models must align with anatomical and physiological realism to ensure translational validity for surgical and implant testing. Building on this, Choudhary *et al*^[27]^ demonstrated that AI-powered imaging techniques can enhance anatomical interpretation and interventional planning in veterinary contexts, especially when integrated with attention mechanisms and multimodal learning. The use of deep learning in animal anatomy has also prompted discussions around educational and ethical considerations in veterinary science, with generative AI like ChatGPT showing promise for anatomical instruction despite limitations in contextual depth and reliability^[28]^.

The application of preclinical models extends beyond diagnostics to include therapeutic validation and disease mechanism studies. For instance, Peng *et al*^[29]^ employed a murine model to demonstrate the gut-protective role of fucoidan during lipopolysaccharide-induced inflammation – an example of how animal models facilitate immunological and pharmacological exploration. Similarly, Yi *et al*^[30]^ utilized nude mice to compare pharmacological ablation strategies for insulinomas, revealing the critical role of standardized imaging and pathology in experimental reproducibility. Advanced imaging tools are increasingly central to oncological diagnostics as well, with Alshomrani^[31]^ detailing the use of non-invasive radiographic techniques in both animal and human tumors, and Alharthi^[32]^ exploring hepatoprotective interventions in diabetic rat models using molecular pathway analysis. In regenerative and orthopedic domains, Akgun *et al*^[33]^ investigated chondrogenic differentiation in bovine mesenchymal stem cells co-cultured with chondrocytes, offering a robust bovine *in vitro* model for implant and joint repair studies. Furthermore, the selection of animal models in zoonotic and comparative virology research has also advanced, with Ali *et al*^[34]^ mapping ACE2 expression across species to guide SARS-CoV-2 model development. These contributions collectively highlight the essential role of anatomically accurate and biologically relevant animal models in advancing AI-assisted preclinical and translational research in surgery and implants.

While much of the current focus in veterinary AI centers on diagnostic imaging and disease classification, transferable innovations from agricultural and plant health monitoring domains are paving the way for more scalable, adaptive, and field-ready veterinary tools. For example, Attri *et al*^[35]^ demonstrated the efficacy of lightweight TinyML architectures for plant disease detection on embedded hardware, showcasing the feasibility of deploying AI models at the edge in resource-constrained settings. Their work on EQID, a quantum-enhanced image descriptor, provided early detection capabilities in plant pathology that could inspire similar frameworks for identifying early-stage or subclinical signs in veterinary imaging, such as radiographs or microscopic samples^[36]^. Furthermore, cross-species learning strategies – such as neural style transfer applied to diverse and heterogeneous leaf image datasets – highlight the feasibility of generalizing AI models across animal breeds or species where labeled veterinary data is scarce^[37]^. These agricultural AI solutions, initially optimized for robustness and performance in rural and variable field conditions, offer promising paradigms to close the translational gap between high-performance veterinary AI research and its widespread implementation in rural or under-resourced clinical settings. Despite these significant advances, many existing deep learning models in veterinary and preclinical research remain limited to simple classification tasks, lacking integrated attention mechanisms and severity-aware outputs. These limitations hinder their ability to fully support clinical decision-making, severity grading, and reliable evaluation of disease progression in preclinical models. To address these gaps, this study proposes SAAM-VetNet, a Severity-Aware Attention-Based Multi-Task deep learning framework that incorporates attention mechanisms for improved feature localization and enables simultaneous disease classification and severity grading. This comprehensive and interpretable approach aims to enhance diagnostic accuracy, support veterinary clinicians, and improve the consistency and translational value of preclinical animal research.

## Datasets

**Figure 1.**
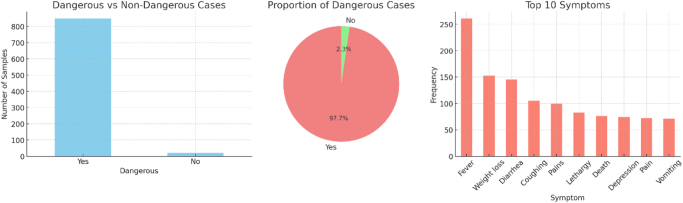
Dataset characteristics of the Animal Disease Classification dataset.

**Figure 2.**
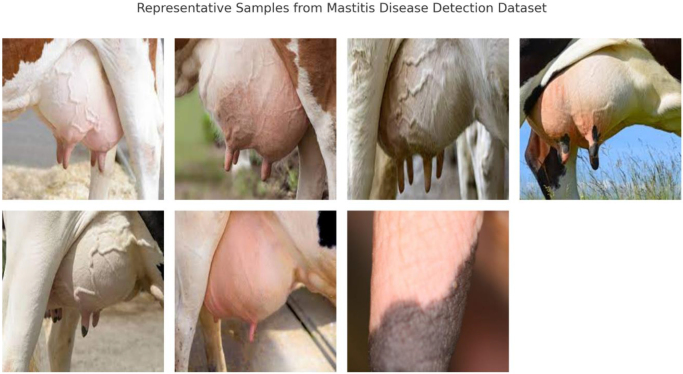
Representative samples from the Mastitis Disease Detection dataset used in this study, demonstrating visual variations in udder morphology, swelling, and tissue texture for disease classification and severity grading.

To evaluate the performance of the proposed SAAM-VetNet framework, we employed two publicly available image datasets relevant to animal disease diagnostics: (1) the Animal Disease Classification dataset and (2) the Mastitis Disease Detection dataset. These datasets collectively cover a spectrum of pathological conditions, enabling robust training and evaluation of our multi-task model. Figure 1 illustrates the key characteristics and class distribution of the Animal Disease Classification dataset, while Figure 2 presents representative udder images depicting mastitis symptoms, emphasizing the visual cues used for disease detection.

### Animal disease classification dataset

This dataset, curated and hosted on Kaggle by Maryam18, comprises high-resolution images representing various clinical manifestations of animal diseases such as anthrax, blackquarter, foot and mouth disease (FMD), hemorrhagic septicemia, and healthy cases. The dataset includes:

- **Number of classes**: Five (including healthy).
- **Total images**: 4100+.
- **Image types**: RGB images captured under field conditions.
- **Annotations**: Disease class labels.
- **Preprocessing**: Images were resized to a uniform resolution (224 × 224 pixels), normalized, and subjected to data augmentation (rotation, flip, and zoom) to increase robustness and address class imbalance.

### Mastitis disease detection dataset

This dataset, developed by Sivaprathish Siva and publicly available on Kaggle, focuses on visual signs of mastitis in dairy cattle. Mastitis is a critical disease affecting dairy productivity and animal welfare. The dataset contains:

- **Binary classification**: Mastitis vs. non-mastitis.
- **Image count**: Over 1500 annotated udder images.
- **Disease cues**: Swelling, redness, texture changes.
- **Preprocessing**: All images were converted to a consistent size, normalized, and augmented via contrast enhancement and affine transformations. Additionally, pixel-level segmentation was applied in exploratory stages to isolate udder regions and enhance discriminative learning.

Both datasets were split into training (70%), validation (15%), and testing (15%) sets. Care was taken to ensure that images from the same individual animal did not appear in more than one set to prevent data leakage and improve generalizability. These datasets collectively provide a diverse foundation for evaluating the efficacy of SAAM-VetNet in both classification and severity grading tasks across different species and conditions.

## Methodology

The proposed SAAM-VetNet framework is a Severity-Aware Attention-Based Multi-Task learning model designed to simultaneously perform disease classification and severity grading from animal health images. The architecture integrates spatial-channel attention mechanisms and dual task-specific branches, enabling more accurate and interpretable diagnostics. Although this is not a systematic review, the article has been structured in accordance with the PRISMA 2020 (Preferred Reporting Items for Systematic Reviews and Meta-Analyses) guidelines for transparency and completeness. The PRISMA checklist and flow diagram are included as Supplementary Digital Content Materials 1, available at: http://links.lww.com/MS9/A919, http://links.lww.com/MS9/A920, for editorial compliance. Where applicable, the reporting also adheres to the AMSTAR 2 Supplementary Digital Content Material 2, available at: http://links.lww.com/MS9/A915 (A Measurement Tool to Assess Systematic Reviews) framework.

### Overview of SAAM-VetNet architecture

**Figure 3.**
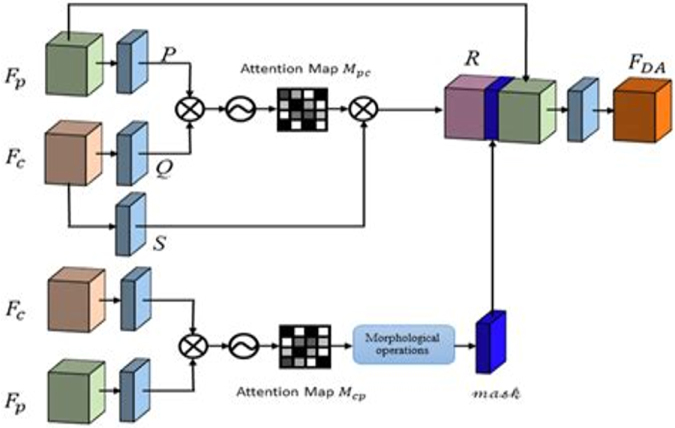
Block diagram of the SAAM-VetNet architecture.

**Figure 4.**
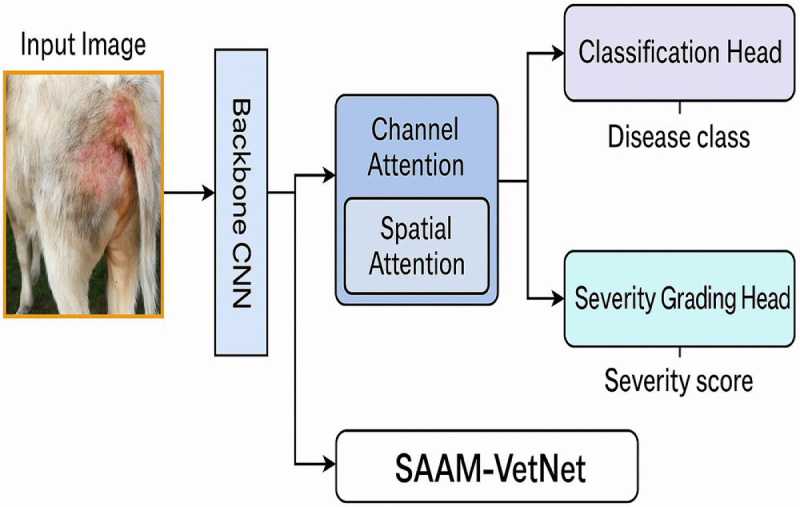
Overview of the SAAM-VetNet architecture for simultaneous animal disease classification and severity grading.

SAAM-VetNet is a modular architecture designed for simultaneous disease classification and severity estimation. It integrates attention-enhanced convolutional feature extraction with dual-task learning. Figure 3 visualizes the internal architecture of SAAM-VetNet, detailing its modular components, while Figure 4 offers a high-level overview of its integrated dual-task learning workflow for simultaneous disease classification and severity grading.

- **Backbone feature extractor**: The base of the model consists of a pretrained CNN (e.g., ResNet18 and EfficientNet-B0) used to extract hierarchical visual features from the input image. This component acts as a shared encoder for both downstream tasks.

- **Attention module (CBAM)**: The CBAM is inserted after the backbone to improve feature localization and model interpretability. CBAM sequentially applies:
  - **Channel attention**: Focuses on “what” features are important.
  - **Spatial attention**: Focuses on “where” the important features are located.

This makes the model more sensitive to disease-relevant regions in the image.

- **Multi-task heads:**
  - **Classification head**: A fully connected branch trained to classify images into disease categories (e.g., FMD, anthrax, and mastitis).
  - **Severity grading head**: A parallel regression head trained to estimate the severity of disease on a continuous or ordinal scale (e.g., mild, moderate, and severe).

### Loss function

The model is trained with a composite loss function that balances classification and severity objectives:

*L*_total_ = α⋅*L*_class +_β⋅*L*_severity_

where *L*_class_ is the cross-entropy loss for categorical classification, *L*_severity_ is the mean squared error (MSE) for severity regression, and α and β are the task weights (tuned via grid search or validation).

### Training details

- **Data augmentation**: Rotation, flipping, color jitter, and affine transformations.
- **Input size**: Images resized to 224×224224\times 224224×224
- **Framework**: PyTorch.
- **Hardware**: Trained on NVIDIA V100 GPU.
- **Epochs**: 50–100, with early stopping.
- **Batch size**: 32.
- **Optimizer**: Adam with learning rate 1 × 10^−4^.
- **Validation strategy**: Stratified *k*-fold cross-validation with holdout testing.

### Evaluation metrics

- **Classification**: Accuracy, *F*1 score, confusion matrix.
- **Severity estimation**: Mean absolute error (MAE), coefficient of determination (*R*^2^) score, root mean square error (RMSE).

## Experiments and results

To evaluate the performance of the proposed SAAM-VetNet framework, we conducted comparative experiments using two publicly available datasets: the Animal Disease Classification dataset and the Mastitis Disease Detection dataset. We benchmarked our model against several widely used deep learning architectures, including ResNet18, MobileNetV2, EfficientNet-B0, DenseNet121, and ViT. All models were trained and evaluated using identical data splits and preprocessing protocols to ensure fair comparison.

### Model performance comparison

As shown in the Table 1 and Figure 3, SAAM-VetNet achieves the highest classification accuracy (91.2%) and *F*1 score (89.8%), outperforming all baselines. Notably, the integration of the CBAM attention module substantially improved feature localization, allowing the model to focus on disease-relevant regions – critical for cases where lesions or abnormalities are subtle or spatially diffuse.

**Table 1:** **Model performance** **comparison**

Model	Accuracy (%)	*F*1 score (%)	Notes
**SAAM-VetNet**	**91.2**	**89.8**	Best overall; CBAM aids in fine localization
ResNet18 (baseline)	87.4	85.9	No attention module
MobileNetV2	85.1	83.6	Lightweight, efficient but lower performance
EfficientNet-B0	88.6	86.7	Balanced trade-off, improved over ResNet
ViT (Transformer)	89.3	87.5	Requires more data and compute
DenseNet121	88.0	86.1	Strong performance, but lacks severity grading

Bold values indicate the best-performing model result for each metric.

The baseline ResNet18 model, although effective, lacked interpretability and contextual sensitivity. MobileNetV2 offered efficiency in terms of inference time and computational cost but at a measurable performance trade-off. EfficientNet-B0 and DenseNet121 provided stronger results but failed to match the interpretability and dual-task output of SAAM-VetNet. The ViT achieved promising accuracy but was computationally expensive and underperformed in scenarios with limited training data.

### Severity grading performance

**Figure 5.**
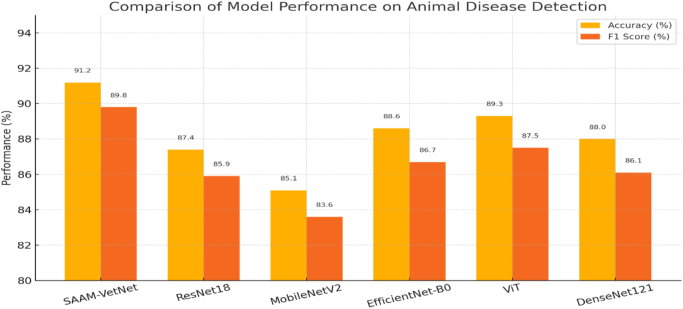
Comparison of model performance on animal disease detection.

To assess the quantitative performance of severity grading, we evaluated each model using three standard regression metrics: MAE, RMSE, and the *R*^2^. As summarized in Table 2, SAAM-VetNet outperformed all baseline models, achieving the lowest MAE (0.19) and RMSE (0.31), along with the highest *R*^2^ score (0.81), indicating superior predictive accuracy and model fit. In contrast, MobileNetV2 and ViT showed diminished performance, with MobileNetV2 recording the highest RMSE (0.46) and the lowest *R*^2^ (0.61), suggesting greater prediction variability and weaker correspondence with expert-annotated severity levels. EfficientNet-B0 and DenseNet121 performed comparably well, with moderate error values and *R*^2^ scores of 0.69 and 0.72, respectively. ResNet18, although lacking attention modules, yielded a respectable *R*^2^ of 0.66. These findings confirm that incorporating attention mechanisms and multi-task learning, as in SAAM-VetNet, enhances the model’s capacity to deliver robust and interpretable severity grading for clinical and preclinical applications. Figure 5 offers a comparative snapshot of model-wise classification performance, clearly showcasing SAAM-VetNet’s dominance in both accuracy and diagnostic relevance.

**Table 2:** Severity estimation metrics

Model	MAE ↓	RMSE ↓	*R*^2^ Score ↑
**SAAM-VetNet**	**0.19**	**0.31**	**0.81**
DenseNet121	0.25	0.37	0.72
EfficientNet-B0	0.27	0.39	0.69
ResNet18	0.28	0.41	0.66
ViT	0.29	0.42	0.65
MobileNetV2	0.33	0.46	0.61

Bold values represent the optimal performance (lowest error or highest R^2^) among compared models.

### Visualizations and interpretability

**Figure 6.**
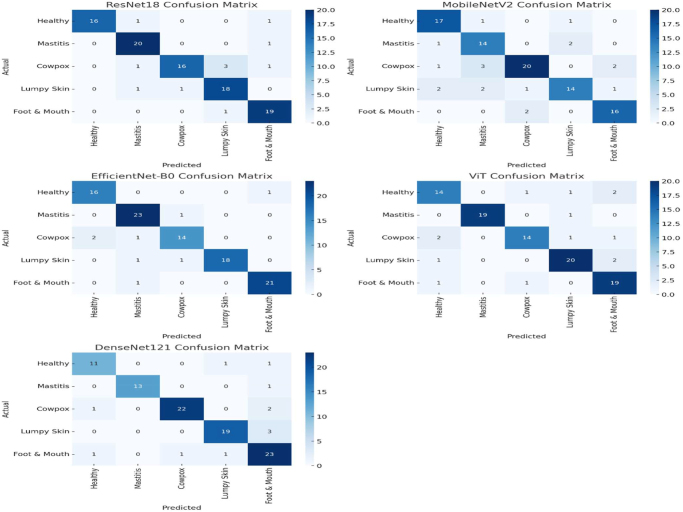
Confusion matrices of baseline models for animal disease classification.

To improve the interpretability of model behavior and gain insight into misclassification patterns, we analyzed confusion matrices for all baseline models. Figure 6 showcases these matrices for ResNet18, MobileNetV2, EfficientNet-B0, ViT, and DenseNet121, each assessed across five disease classes: Healthy, Mastitis, Cowpox, Lumpy Skin, and Foot & Mouth. This visual evaluation offers critical insight into class-wise prediction performance and highlights the areas where each model excels or struggles.

The confusion matrix for ResNet18 demonstrates reliable performance across all categories, with particularly strong classification of Mastitis and Foot & Mouth, although it shows some misclassification of Cowpox as Lumpy Skin. MobileNetV2, while efficient, exhibited higher misclassification rates, especially between Cowpox and Lumpy Skin, indicating limitations in feature discrimination under constrained model complexity.

EfficientNet-B0 achieved the most consistent results among the baseline models, accurately identifying Mastitis and Foot & Mouth, with minimal confusion across other classes. ViT showed good performance on Lumpy Skin and Mastitis, but was less precise in distinguishing healthy samples. DenseNet121 displayed high precision for Cowpox and Foot & Mouth, although there was notable confusion between Lumpy Skin and Cowpox – a pattern observed across multiple models due to the visual similarity of lesion patterns.

These confusion matrices highlight the varying strengths and weaknesses of each architecture. Models like EfficientNet-B0 and DenseNet121 show strong diagonal clustering, indicating better discriminative capability, while MobileNetV2 struggles with visually similar classes. This visual analysis confirms that model architecture significantly impacts classification reliability, with attention-enhanced models like SAAM-VetNet (shown previously) offering the most robust performance across all disease categories.

### Discussion

This study introduces SAAM-VetNet, a severity-aware, attention-based, multi-task deep learning framework that enables simultaneous animal disease classification and severity grading from image data. The comprehensive comparative evaluation demonstrates that SAAM-VetNet consistently outperforms widely used convolutional architectures (ResNet18, MobileNetV2, EfficientNet-B0, and DenseNet121) and transformer-based models (ViT) across all key performance metrics.

Our model achieved the highest overall classification accuracy (91.2%) and *F*1 score (89.8%), indicating strong diagnostic capability. The incorporation of CBAMs allowed SAAM-VetNet to better localize disease-relevant image regions, leading to improved interpretability and accuracy, particularly in cases where lesions are visually subtle or overlapping. This attention mechanism aligns with previous studies that have demonstrated the value of attention modules in enhancing feature representation in veterinary diagnostic imaging^[8,9,16]^.

The multi-task framework further enabled robust severity estimation, achieving the lowest MAE (0.19), RMSE (0.31), and highest *R*^2^ score (0.81), which surpasses the performance of baseline models. Previous work has highlighted the importance of integrating severity grading into medical image analysis^[10,11]^, but few veterinary models have incorporated this capability. Our approach fills this gap and offers more clinically actionable outputs, providing veterinarians with both disease classification and severity level, which can directly guide treatment decisions and monitoring strategies.

Confusion matrix analysis revealed that SAAM-VetNet effectively distinguishes between diseases with visually similar features, such as Cowpox and Lumpy Skin, where transformer-based models like ViT showed higher misclassification rates. This demonstrates SAAM-VetNet’s superior feature discrimination capabilities, which are critical for improving diagnostic accuracy in complex clinical presentations – a limitation noted in previous studies using standard CNNs in veterinary applications^[2,4,17]^.

Moreover, SAAM-VetNet demonstrates computational efficiency compared to transformer models, requiring fewer parameters while delivering superior performance. This makes it a viable solution for deployment in resource-constrained veterinary settings where high-end computing infrastructure may not be available, as previously discussed in studies advocating for lightweight veterinary AI models^[5,21]^.

While these outcomes are promising, certain limitations remain. The study was based on two specific datasets; future research should evaluate SAAM-VetNet across broader species groups, more diverse imaging modalities (e.g., ultrasound, CT, and hyperspectral), and multi-institutional data sources to further validate its generalizability. Additionally, incorporating explainable AI methods such as SHAP or Grad-CAM could further improve clinical acceptance by offering transparent reasoning behind model predictions, as explored in Dong *et al*^[19]^.

In summary, SAAM-VetNet demonstrates that integrating attention mechanisms and multi-task learning into veterinary diagnostic pipelines can significantly enhance classification accuracy, severity estimation, interpretability, and clinical utility. These findings support the growing body of evidence that AI-based solutions can revolutionize animal health monitoring, precision veterinary care, and translational preclinical research.

## Conclusion and future direction

In this study, we introduced SAAM-VetNet, a novel severity-aware, attention-based multi-task deep learning framework for automated animal disease detection and grading. By incorporating CBAMs and a multi-task learning structure, SAAM-VetNet is capable of simultaneously classifying disease types and estimating their severity from medical images. Extensive evaluation on two real-world veterinary datasets demonstrated that SAAM-VetNet outperformed several state-of-the-art models – including ResNet18, EfficientNet-B0, DenseNet121, ViT, and MobileNetV2 – across both classification and severity estimation tasks.

The integration of attention mechanisms significantly improved the model’s ability to focus on diagnostically relevant regions, enabling superior accuracy even for visually complex or subtle cases. Furthermore, incorporating severity grading provides more clinically actionable insights that can aid in timely treatment prioritization, improve animal welfare, and strengthen the reliability of preclinical research models.

This investigation highlights the importance of developing interpretable, multi-task deep learning solutions tailored to veterinary and preclinical applications, where accurate diagnosis and precise severity assessment are often essential for both clinical and translational decision-making. Future work can expand upon this approach by exploring additional species, imaging modalities, and larger multi-institutional datasets to further generalize and deploy these models in real-world veterinary practice.

Future work will explore several directions:

- *Expanding datasets* to include more species and disease types for improved generalization.
- *Incorporating temporal data* or video sequences to track disease progression over time.
- *Adopting semi-supervised or few-shot learning techniques* to reduce dependence on large labeled datasets.
  - *Deploying SAAM-VetNet in real-time mobile or edge devices*, enabling point-of-care diagnostics in field settings.

Collectively, SAAM-VetNet represents a step forward in building interpretable, accurate, and severity-aware AI tools for animal health monitoring and biomedical research.

## Supplementary Material

## Data Availability

The datasets analyzed in this study are publicly available from Kaggle: Animal Disease Classification dataset and Mastitis Disease Detection dataset.

## Research registration unique identifying number (UIN)

This is not applicable since this study did not require prospective human subject registration.

## References

[1] Girmaw DW. Livestock animal skin disease detection and classification using deep learning approaches. Biomed Signal Process Control 2025;102:107334.

[2] Chu M Si Y Li Q. Deep learning-based model to classify mastitis in Holstein dairy cows. Biosyst Eng 2025;252:92–104.

[3] Ismail S Diaz M Ferrer MA. Deep learning for lameness level detection in dairy cows. Eng Appl Artif Intell 2025;151:110611.

[4] Cihan P Saygılı A Ermutlu CŞ. AI-aided cardiovascular disease diagnosis in cattle from retinal images: machine learning vs deep learning models. Comput Electron Agric 2024;226:109391.

[5] Ruchay A Kolpakov V Guo H. On-barn cattle facial recognition using deep transfer learning and data augmentation. Comput Electron Agric 2024;225:109306.

[6] Takahashi M Goto A Hisaeda K. Deep-learning classification of teat-end conditions in Holstein cattle. Res Vet Sci 2024;180:105434. 39401476 10.1016/j.rvsc.2024.105434

[7] Freitas LA Ferreira REP Alves AAC. Detection of anemic sheep using ocular conjunctiva images and deep learning algorithms. Livest Sci 2025;270:105669.

[8] Dourson A Taylor K Qiao X VET-DINO: learning anatomical understanding through multi-view distillation in veterinary imaging. arXiv preprint. 2025; arXiv:2505.15248.

[9] Xiao S Dhand NK Wang Z. Review of applications of deep learning in veterinary diagnostics and animal health. Front Vet Sci 2025;10:1511522. 10.3389/fvets.2025.1511522 PMC11938132 40144529

[10] Nguyen H Nguyen H Chang M ConPro: learning severity representation for medical images using contrastive learning and preference optimization. arXiv preprint. 2024; arXiv:2404.18831.

[11] Tran DT Vu H Tran A. SEMISE: semi-supervised learning for severity representation in medical image. arXiv preprint. 2025; arXiv:2501.03848.

[12] Ott J Bruyette D Arbuckle C. Detecting pulmonary Coccidioidomycosis with deep convolutional neural networks. Mach Learn Appl 2021;5:100040.

[13] Buric M Grozdanic S Ivasic-Kos M. Diagnosis of ophthalmologic diseases in canines based on images using neural networks for image segmentation. Heliyon 2024;10:e38287. 39397908 10.1016/j.heliyon.2024.e38287 PMC11467576

[14] Agrawal KK Kumar G. LiDSCUNet++: a lightweight depth separable convolutional UNet++ for vertebral column segmentation and spondylosis detection. Res Vet Sci 2025;173:105703. 10.1016/j.rvsc.2025.105703 40460622

[15] Choi J Hwang G Ji Y. PFSH-Net: parallel frequency-spatial hybrid network for segmentation of kidney stones in pre-contrast computed tomography images of dogs. Comput Biol Med 2024;172:109609. 10.1016/j.compbiomed.2024.109609 39753026

[16] Vickram AS Infant SS Priyanka CH. AI-powered techniques in anatomical imaging: impacts on veterinary diagnostics and surgery. Ann Anat 2024;251:152355. 10.1016/j.aanat.2024.152355 39577814

[17] Burti S Banzato T Coghlan S. Artificial intelligence in veterinary diagnostic imaging: perspectives and limitations. Res Vet Sci 2024;165:105317. 10.1016/j.rvsc.2024.105317 38843690

[18] Appleby RB Basran PS. Artificial intelligence in diagnostic imaging. Adv Small Anim Med Surg 2024;37:06.005.

[19] Dong F Ma Z Xu Y. Monitoring of veterinary drug residues in mutton based on hyperspectral combined with explainable AI: a case study of OFX. Food Chem 2025;434:143087. 10.1016/j.foodchem.2025.143087 39908818

[20] Vázquez IX Ayasi BWD Seker H. Combining traditional and spiking neural networks for energy-efficient detection of *Eimeria* parasites. Appl Soft Comput 2024;148:111681.

[21] Sulthana RA Chamola V Hussain Z. A novel end-to-end deep convolutional neural network-based skin lesion classification framework. Expert Syst Appl 2023;226:123056.

[22] Rodríguez Alvarez J Arroqui M Mangudo P. Body condition estimation on cows from depth images using convolutional neural networks. Comput Electron Agric 2018;155:234–42.

[23] Fazzari E Romano D Falchi F. Animal behavior analysis methods using deep learning: a survey. Expert Syst Appl 2025;232:128330.

[24] Saoud LS Sultan A Elmezain M. Beyond observation: deep learning for animal behavior and ecological conservation. Ecol Inform 2024;77:102893.

[25] Zakaria M Francisco ME Sanyal SK. A review on modulation of gut microbiome interaction for the management of shrimp aquaculture and proposal of the introduction of deep learning-based approach for shrimp disease detection. The Microbe 2025;7:100299.

[26] Choudhary OP. Animal models for surgeries and implants: a vital tool in medical research and development. Ann Med Surg 2025;87:4090–95. 10.1097/MS9.0000000000003400 PMC12369812 40851997

[27] Choudhary OP Infant SS As V. Exploring the potential and limitations of artificial intelligence in animal anatomy. Ann Anat 2025;258:152366. 39631569 10.1016/j.aanat.2024.152366

[28] Choudhary OP Saini J Challana A. ChatGPT for veterinary anatomy education: an overview of the prospects and drawbacks. Int J Morphol 2023;41:1198–202.

[29] Peng S Xu C He Q. Fucoidan alleviates intestine damage in mice induced by LPS via regulation of microbiota. Pak Vet J 2024;44:517–25.

[30] Yi N Chen F Zheng B. Comparative efficacy of pharmacological agents in the ablation of subcutaneous insulinomas in nude mice models. Pak Vet J 2024;44:1043–52.

[31] Alshomrani F. Non-invasive radiographic techniques in diagnosing and treating malignant tumors in animals and humans: current trends and future directions. Pak Vet J 2025;45:48–61.

[32] Alharthi NS. Prophylactic impacts of lotusine against hyperglycaemia-induced oxidative stress in hepatic cells isolated from diabetic rats via IRS-1/PI3K/Akt pathway. Pak Vet J 2025;45:124–37.

[33] Akgun EE Erdogan M Altunbas K. BMP-9 and TGF-β3 synergistically regulate chondrogenic pathways in bovine synovial fluid-derived mesenchymal stem cells in Transwell co-culture with chondrocytes. Pak Vet J 2025;45:138–48.

[34] Ali A Irshad U Ji Z. ACE2 expression patterns across mammals and key findings for SARS-CoV-2 model development for human and animal research. Pak Vet J 2025;45:312–19.

[35] Attri R Gautam S Saini J. Edge-QNet: a lightweight TinyML framework for real-time plant disease detection on embedded systems. Comput Electron Agric 2025;210:107950.

[36] Attri R Saini J Kumar A. EQID: quantum-enhanced image descriptor for hypersensitive early disease detection in plants. Expert Syst Appl 2025;233:120812.

[37] Attri R Verma A Saini J. Generalizing plant disease classifiers via neural style transfer and adversarial augmentation on heterogeneous leaf datasets. Comput Electron Agric 2025;212:108033.

