# Supplementary material for: SAAM-VetNet: an attention-based multi-task framework for animal disease detection and severity grading
Source: Ann Med Surg (Lond). 2025 Aug 19;87(10):6399–408. doi: 10.1097/MS9.0000000000003728 (PMC12577985; doi:10.1097/MS9.0000000000003728)
Supplement: Supplementary file 2 [file ms9-87-6399-s002.docx]

**Note: This flow diagram is included as a formal requirement. The study is an original deep learning investigation, not a systematic review.**

**Identification of studies via databases and registers**

Records removed *before screening*:

Duplicate records removed (n =NA )

Records marked as ineligible by automation tools (n =NA )

Records removed for other reasons (n =NA )

Records identified from*:

Databases (n = NA)

Registers (n = NA)

**Identification**

Records screened

(n = NA)

Records excluded**

(n =NA )

Reports sought for retrieval

(n = NA)

Reports not retrieved

(n = NA)

**Screening**

Reports assessed for eligibility

(n = NA)

Reports excluded:

Reason 1 (n = NA)

Reason 2 (n = NA)

Reason 3 (n = NA)

etc.

Studies included in review

(n =NA )

Reports of included studies

(n = NA)

**Included**

*Consider, if feasible to do so, reporting the number of records identified from each database or register searched (rather than the total number across all databases/registers).

**If automation tools were used, indicate how many records were excluded by a human and how many were excluded by automation tools.

Source: Page MJ, et al. BMJ 2021;372:n71. doi: 10.1136/bmj.n71.

This work is licensed under CC BY 4.0. To view a copy of this license, visit <https://creativecommons.org/licenses/by/4.0/>
